# Supplementary material for: Adaptively introgressed Neandertal haplotype at the OAS locus functionally impacts innate immune responses in humans
Source: Genome Biol. 2016 Nov 29;17:246. doi: 10.1186/s13059-016-1098-6 (PMC5129249; doi:10.1186/s13059-016-1098-6)
Supplement: Additional file 1: — Supplementary figures. (PDF 2303 kb) [file 13059_2016_1098_MOESM1_ESM.pdf]

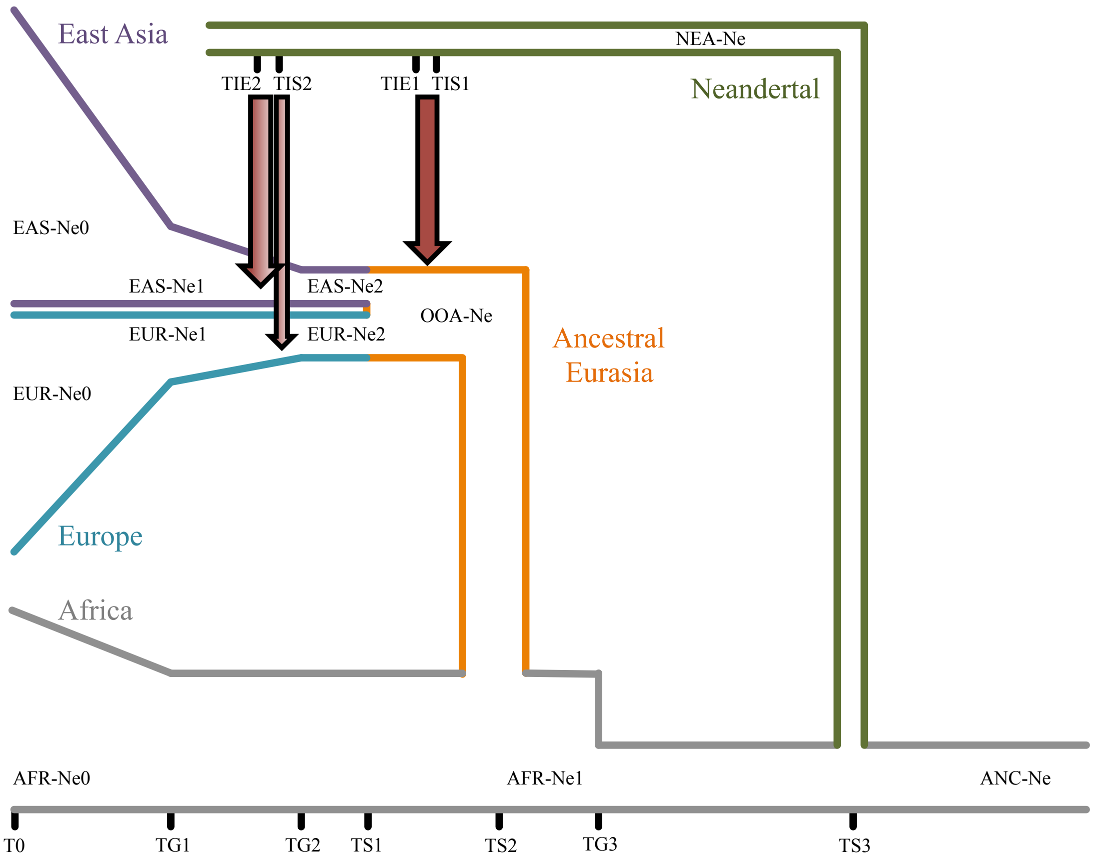

Figure S1: Schematic representation of demographic model used in simulations. Red arrows indicate points of introgression. The full range of demographic parameters used for the simulation can be found in Table S1.

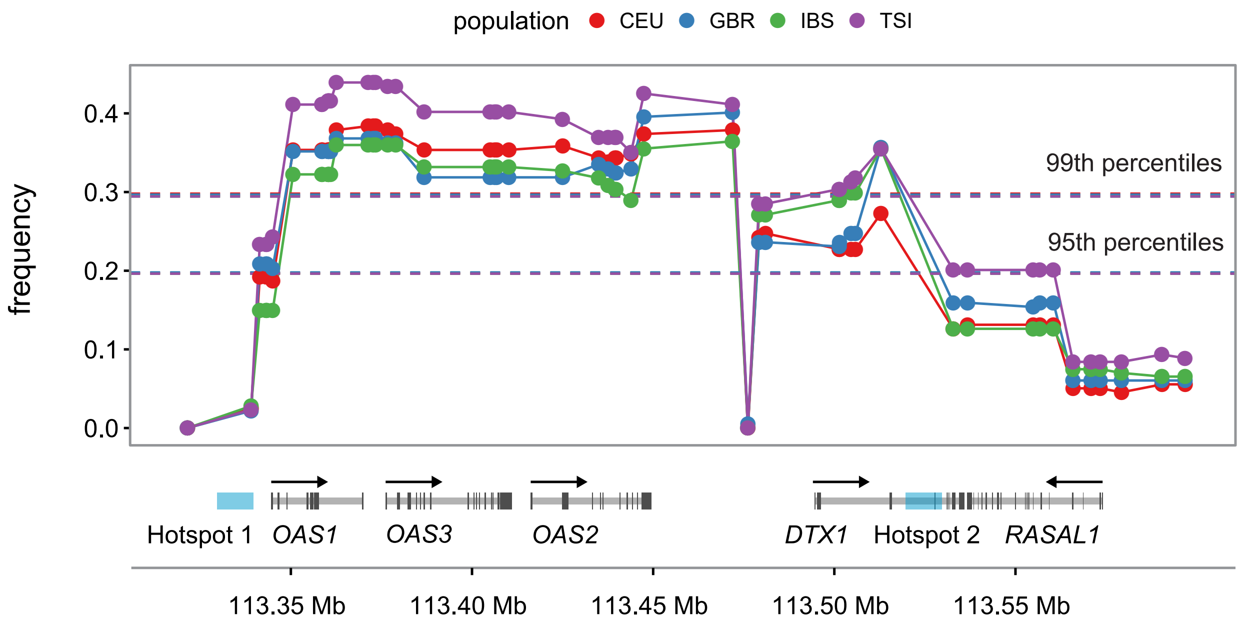

Figure S2: OAS-introgressed haplotypes are found at higher frequencies in European populations than expected under neutrality when using a two-pulse introgression model. (A) Comparison of frequency (y- axis) of NLS in the OAS locus in the CEU, GBR, IBS, and TSI European population samples with respect to neutral expectations (dashed lines) based on coalescent simulations considering two-pulse introgression in both Europeans and Asians, or a second pulse of introgression only in Europeans. Dashed lines overlap almost completely, reflecting little variation between a two-pulse introgression in both European and Asians, or a second pulse only in Europeans.

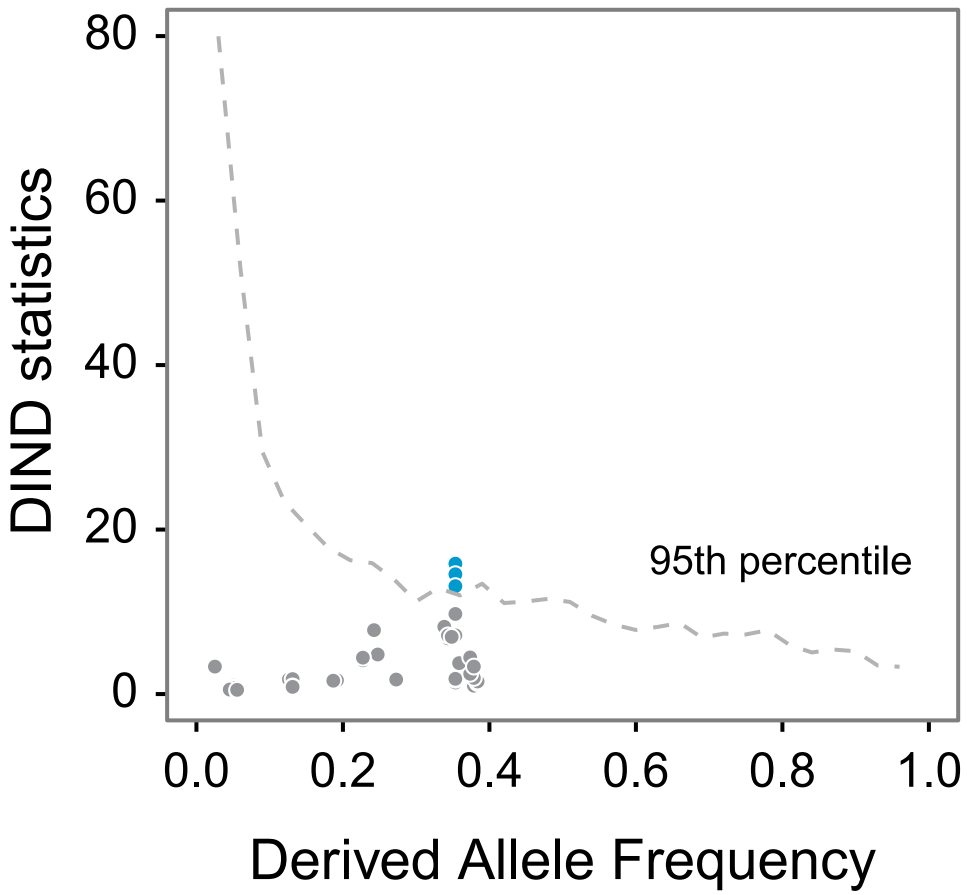

Figure S3: The DIND statistic indicates reduced diversity surrounding NLS. DIND (y-axis) is plotted for SNPs in OAS locus by derived (Neandertal) allele frequency. Dashed line indicates genome-wide 95th percentile by frequency.

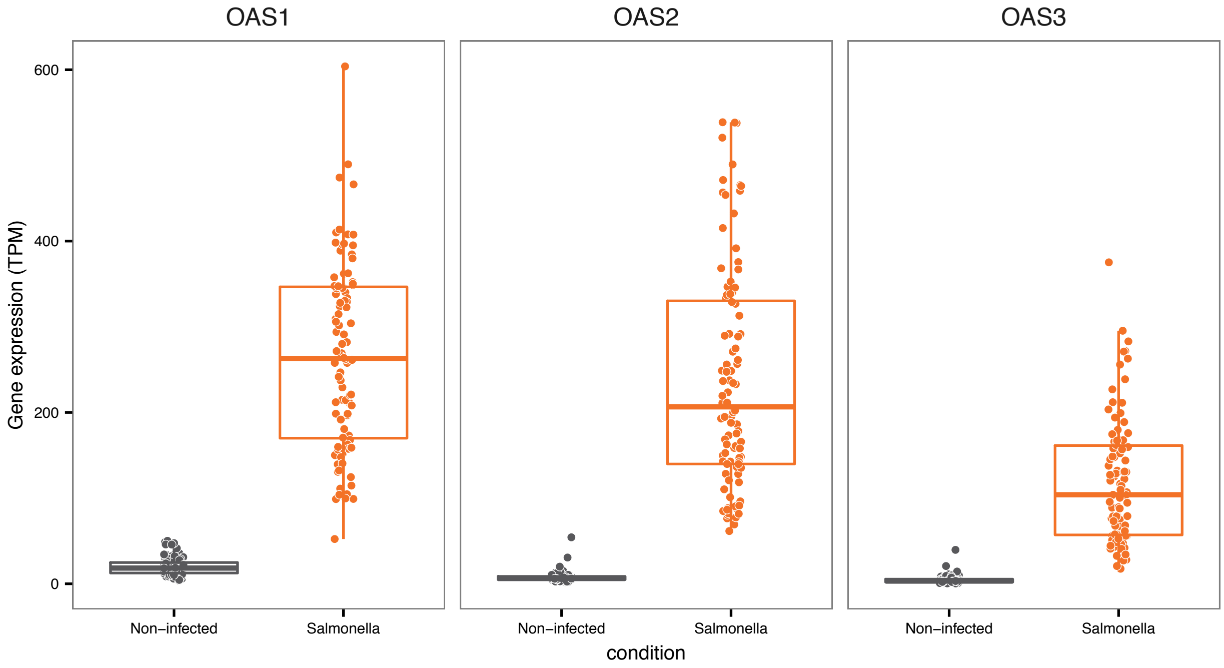

Figure S4: Expression levels of OAS genes in primary macrophages (European) before and after infection with Salmonella.

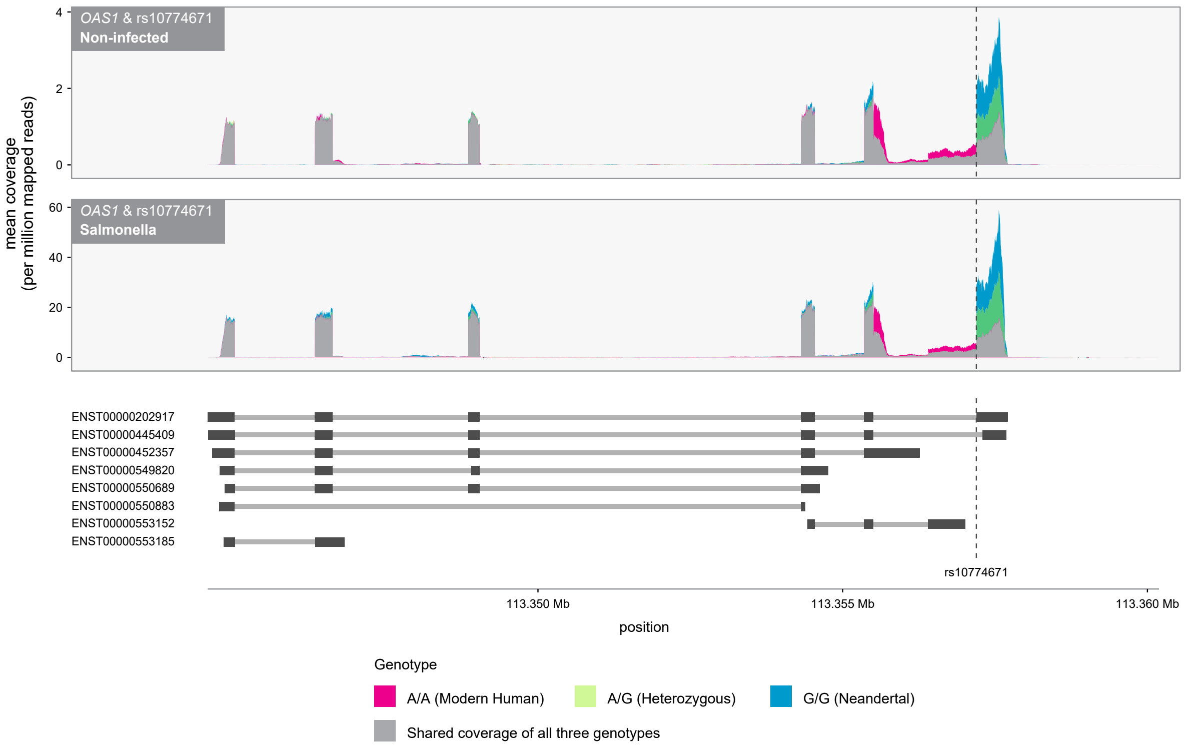

Figure S5: The splicing variant rs10774671 is a strong asQTL for *OAS1.* Plotted is the normalized average coverage at which each base was sequenced along the genomic regions encoding the gene *OAS1*. Individuals were stratified according to their genotype at rs10774671. Below the figure are gene models from the Ensembl database. Individuals carrying the G allele at rs10774671 (i.e. the Neandertal allele) primarily express the transcript ENST00000202917 (referred to as p46 in the text) whereas individuals carrying the A derived allele lose the splice site, which leads to the usage of a distinct isoform.

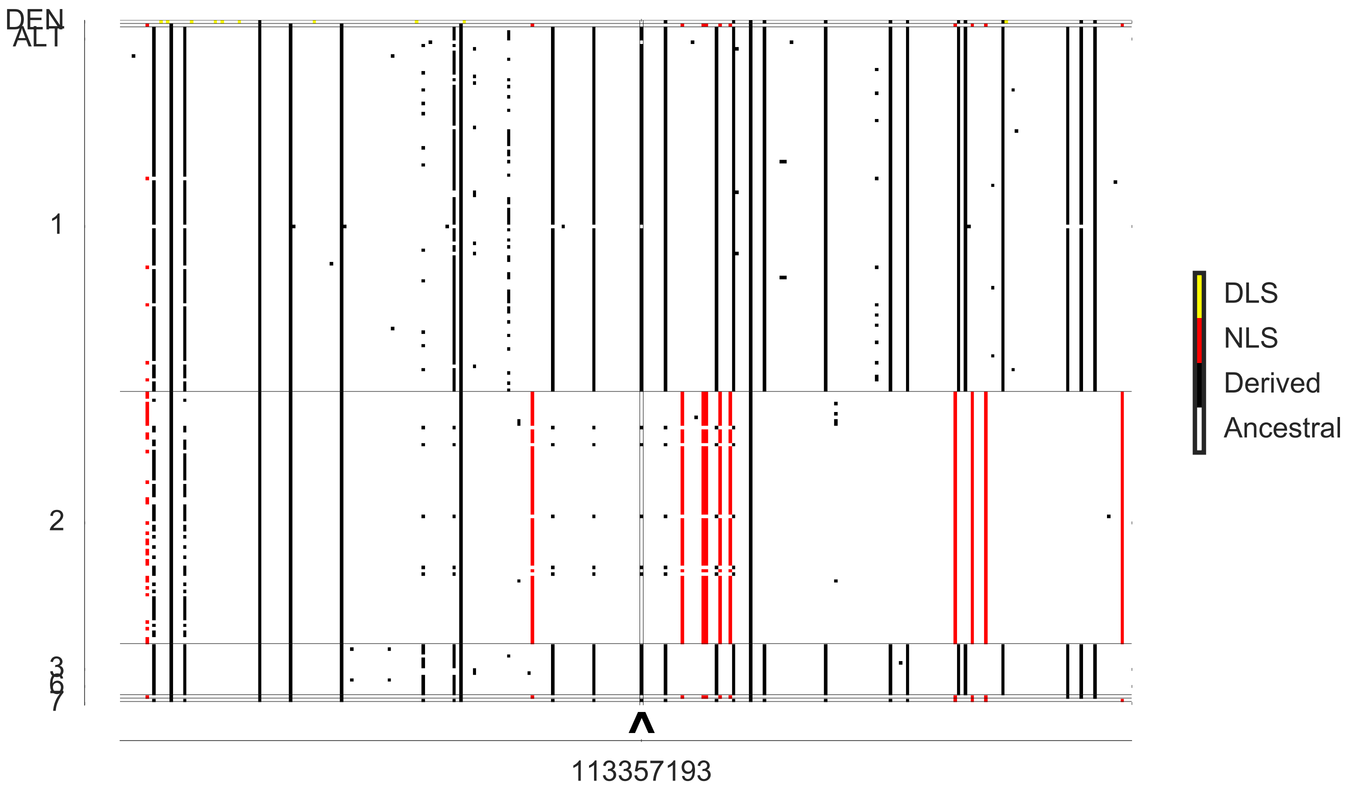

Figure S6: Haplogram of region surrounding *OAS1* splice site (rs10774671). 198 phased haplotypes from the CEU population sample are illustrated along with the Altai and Denisovan haplotypes. Ancestral alleles are colored white, derived alleles are (1) yellow if derived in Denisovan and absent from Africans (YRI) (DLS), (2) red if derived in Neandertals and absent from Africans (YRI) (NLS), (3) black otherwise. With the exception of two rare haplotypes in cluster 1, all occurrences of the ancestral variant at rs10774671 are surrounded by derived Neandertal alleles, indicating that these were introduced in a Neandertal haplotype. Only sites where the Neandertal and Denisovan genomes are homozygous were used in this haplogram. Cluster labels (y-axis) correspond to clusters in Figure 1.

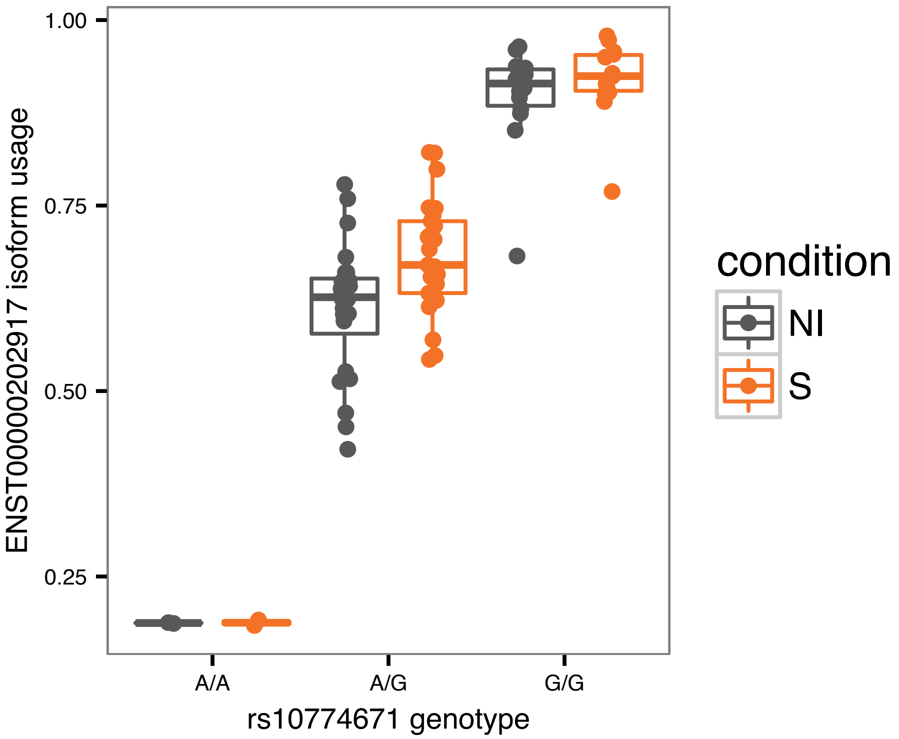

Figure S7: Isoform usage for transcript ENST00000202917 stratified by genotype at rs10774671 in cells derived from individuals with African ancestry. The derived (Neandertal) allele leads to increased expression of ENST00000202917 in both non-infected and *Salmonella* infected conditions.

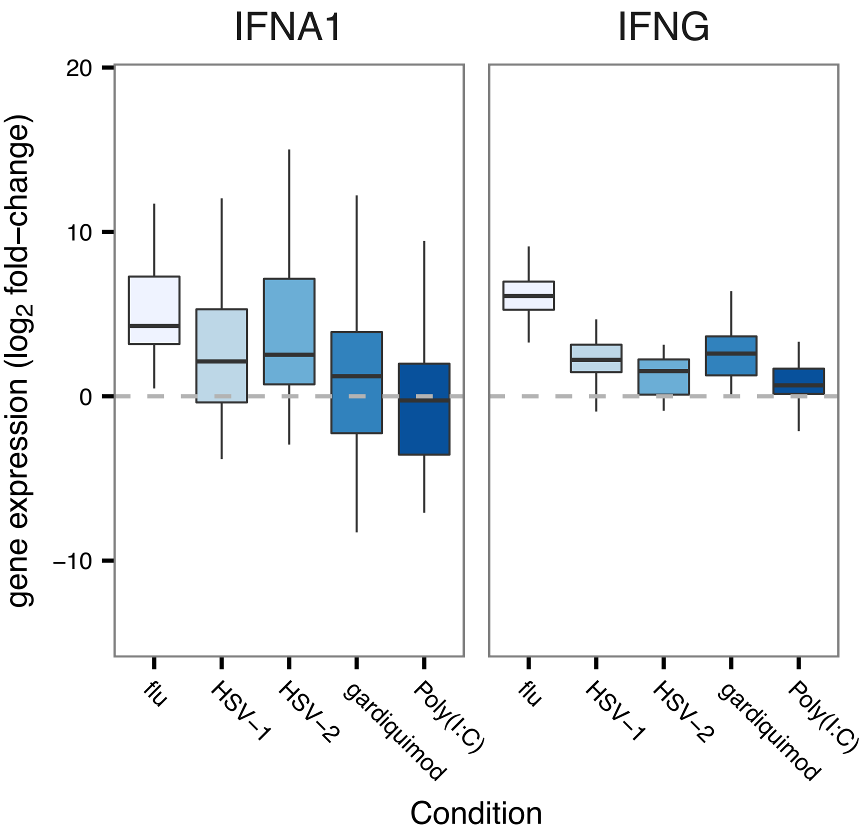

Figure S8: Log 2 fold induction (y-axis) of IFNA1 (type 1 interferon) and IFNG (type II interferon) in PBMCs upon stimulation with several viral agents.

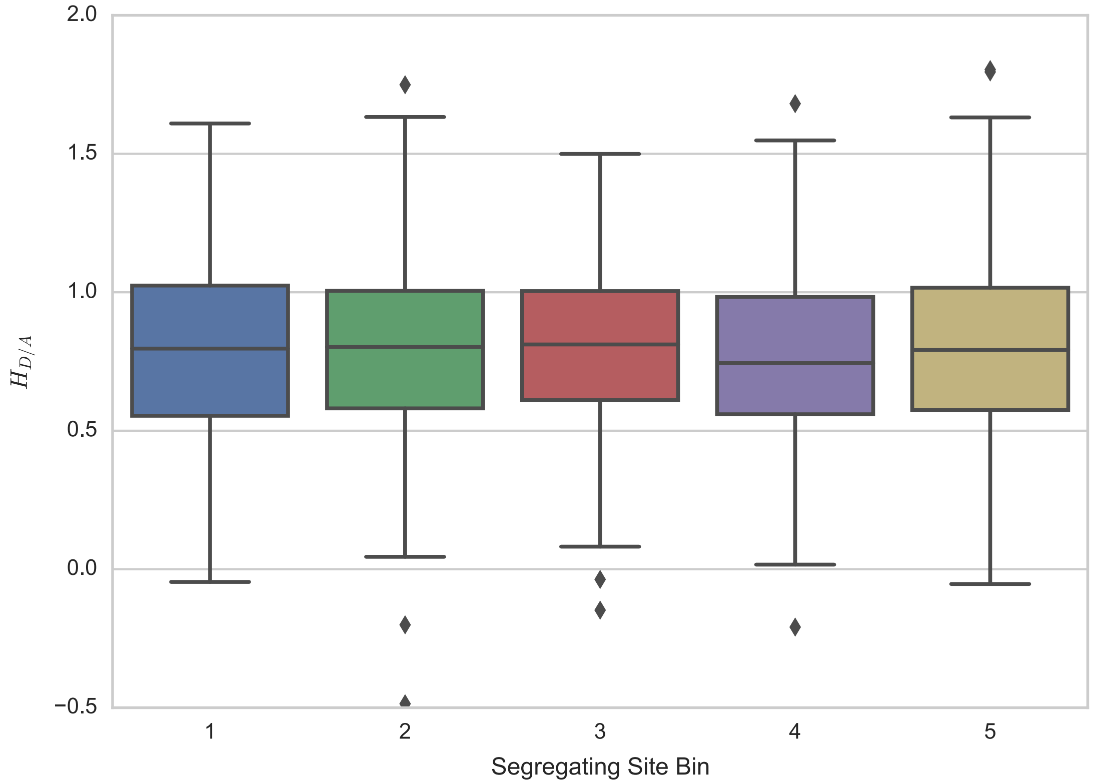

Figure S9: $H_{D/A}$ plotted against quintiles of segregating sites in 1,000 simulations. The $H_{D/A}$ statistic does vary with number of segregating sites across simulated data.

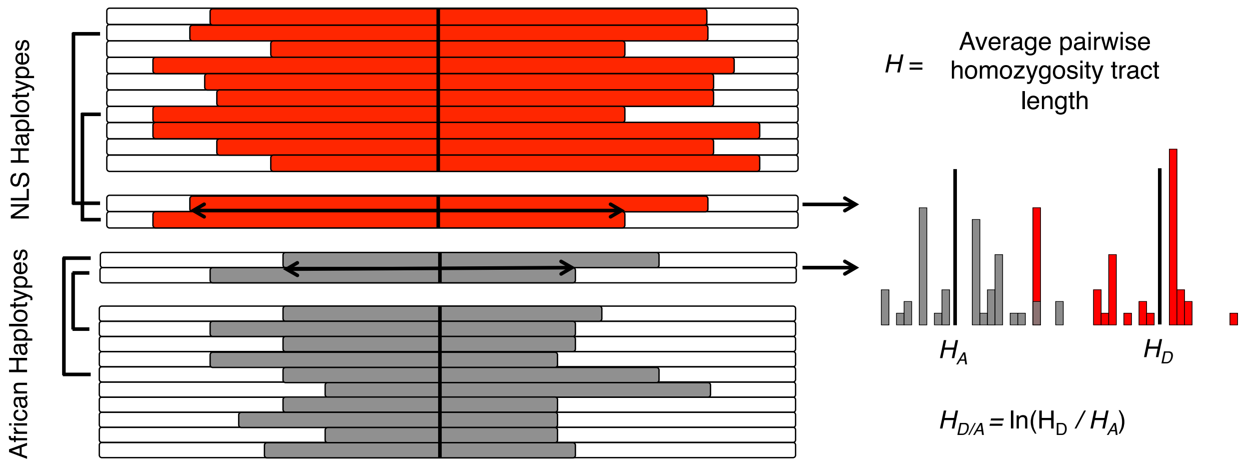

Figure S10: Schematic illustration of the $H_{D/A}$ statistic. At each NLS, haplotypes are divided into two groups, based on whether they carry the Neandertal (derived) or African (ancestral) state. Within each haplotype subset, haplotype homozygosity is calculated across all pairs of haplotypes and then averaged (H), resulting in two values; H_A and H_D (A-ancestral, D-derived). Finally, $H_{D/A}$ is calculated as the natural log of the ratio of derived to ancestral H values. Excessively high values of this statistic reflect particularly long haplotypes carrying Neandertal-derived alleles.

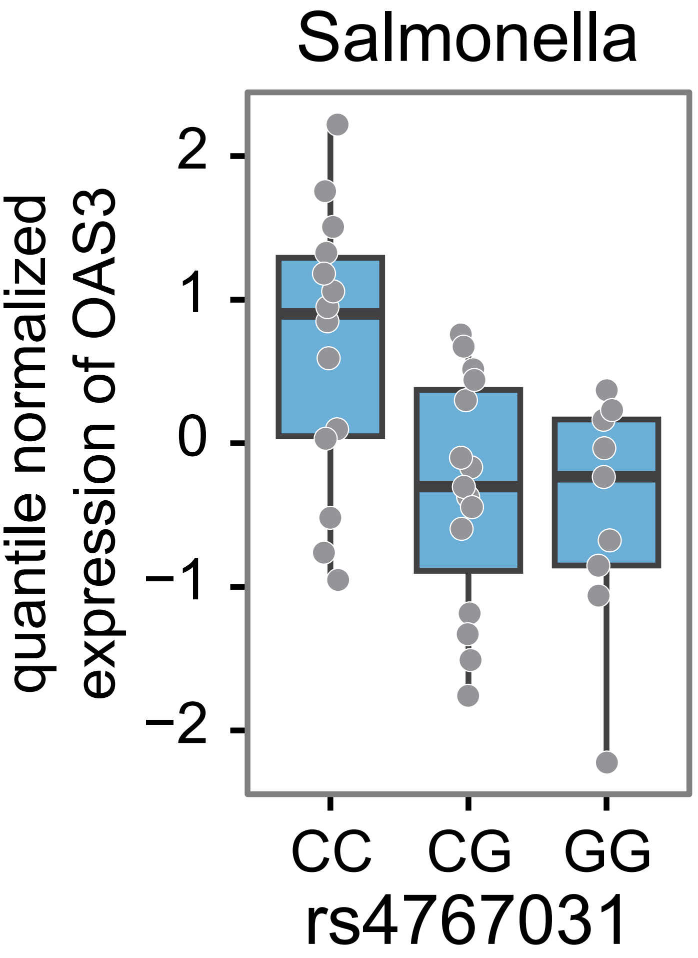

Figure S11: Boxplot for the association between genotypes at the NLS rs4767031 (x-axis) and the expression levels of *OAS3* in *Salmonella*-infected macrophages using real-time PCR.
